# Effects of algal oil as an alternative to fish oil in feline foods on serum concentrations of eicosapentaenoic acid and docosahexaenoic acid

**DOI:** 10.1093/jas/skaf252

**Published:** 2025-09-05

**Authors:** Laura A Motsinger, Catherine R Kalmbach, John Brejda, Nasson Mwakatage, Leslie Hancock, Dale A Fritsch

**Affiliations:** Hill’s Pet Nutrition, Topeka, KS 66617, USA; Hill’s Pet Nutrition, Topeka, KS 66617, USA; Alpha Statistical Consulting, Lincoln, NE 68502, USA; Hill’s Pet Nutrition, Topeka, KS 66617, USA; Hill’s Pet Nutrition, Topeka, KS 66617, USA; Hill’s Pet Nutrition, Topeka, KS 66617, USA

**Keywords:** algal oil, docosahexaenoic acid, eicosapentaenoic acid, feline, fish oil

## Abstract

Fish oil is a source of the n-3 fatty acids eicosapentaenoic acid (EPA) and docosahexaenoic acid (DHA) that confer several health benefits. To ensure continuity in the supply of n-3 fatty acids, alternative sources are being sought. Algal oil may serve as a promising alternative to fish oil for supplementing DHA in cat foods. The purpose of this study was to determine if the inclusion of algal oil in place of fish oil in feline foods would result in similar serum EPA and DHA concentrations. Cats were first fed a control food for 5 wk and then randomized into two groups and fed test foods containing either fish oil or algal oil in sequential increasing concentrations of DHA (0.2, 0.4, and 0.6%). Serum was analyzed at the beginning and end of each of these 5-wk feeding periods. Oil type had no effect on the body weight of cats consuming foods containing either algal oil or fish oil; only cats that consumed the algal oil food with 0.6% DHA had a significant decrease from baseline in food intake (*P* = 0.0011). Analysis of serum fatty acid concentrations showed that serum DHA increased at similar rates when fish oil and algal oil levels were increased in the food. Although increasing levels of fish and algal oil both increased serum EPA concentrations, the higher concentrations of EPA in the fish oil foods resulted in higher circulating concentrations of EPA in those cats. Algal oil was included at levels 3.7-fold lower than fish oil due to the high DHA content of algal oil. Overall, these data indicate that algal oil may serve as a good alternative dietary source of DHA. Fatty acid profiles of algal oil should be considered when selecting a replacement for fish oil in feline foods.

## Teaser

Inclusion of a predominantly DHA-containing algal oil in place of fish oil in feline food provides similar serum bioactivity of DHA to that of fish oil.

## Introduction

The n-3 polyunsaturated fatty acids (PUFAs) such as eicosapentaenoic acid (EPA) and docosahexaenoic acid (DHA) confer several beneficial effects on animal health. In dogs, studies of dietary supplementation with fish oil have demonstrated a reduction of cholesterol and triglycerides in dogs with hyperlipidemia (de Albuquerque et al., 2021), reduced arrhythmia in dogs with right ventricular cardiomyopathy (Smith et al., 2007), renoprotective effects in dogs with renal insufficiency (Brown et al., 1998), improvements in osteoarthritis (Fritsch et al., 2010; Roush et al., 2010; Mehler et al., 2016), and improved cognitive function in puppies (Zicker et al., 2012; Rodrigues et al., 2023). Fish oil supplementation in cats has shown improvements in mobility in those with osteoarthritis (Corbee et al., 2013) or degenerative joint disease (Lascelles et al., 2010), reduced risk of urinary stone formation (Hall et al., 2017), improved glucose control and hyperinsulinemia (Wilkins et al., 2004), shown cognitive enhancement (Pan et al., 2013), increased leukocyte phagocytic activity and T-cell function (Rutherfurd-Markwick et al., 2013), and reduced skin inflammatory responses (Park et al., 2011). These studies used various concentrations and ratios of DHA and EPA as well as different lengths of feeding time periods and disparate outcome measures, so it is not possible to state a DHA or EPA concentration at which effects can be observed.

Due to limited ability to synthesize long-chain PUFAs, cats require dietary EPA and DHA (Rivers et al., 1975; Morris, 2002). The global demand for these n-3 PUFAs has increased due to the consumption of EPA and DHA supplements by humans and their incorporation into companion animal food, most commonly as fish oil. However, fisheries worldwide cannot currently meet the demand for fish oil (Jenkins et al., 2009; Oils and Fats International, 2024), underscoring the importance of establishing alternative sources of n-3 fatty acids in order to reduce reliance on fish harvests. Fish do not synthesize EPA and DHA de novo but rather bioaccumulate them by consuming plankton and algae (Todorčević and Hodson, 2015). Thus, algal oil appears to be a promising and viable sustainable alternative to fish-based oils.

While there are no recommended adult maintenance minimum or maximum levels for EPA or DHA from the American Association of Feed Control Officials (AAFCO) or European Pet Food Industry Federation (FEDIAF) (AAFCO, 2024; European Pet Food Industry Federation (FEDIAF), 2024), the safety of pet foods containing up to 3% algal oil (1.62% EPA + DHA) on a dry matter basis has been demonstrated in dogs (Dahms et al., 2019) and cats (Vuorinen et al., 2020) during gestation, lactation, and growth until 26 to 32 wk of age. No changes in hematology, blood chemistry, or coagulation parameters were reported in these dogs and cats fed the algal oil-enriched foods when compared to a control food. The U.S. Food and Drug Administration deemed the microalgal oil utilized in the present study and others (Dahms et al., 2019; Vuorinen et al., 2020) as generally regarded as safe for use in canned and extruded dog and cat food up to 1.5% on a dry matter basis in 2020 (U.S. Food and Drug Administration, 2025).

The present study examined the effects of providing dietary DHA from fish oil or a predominantly DHA-containing algal oil on food intake, body weight, and serum fatty acid concentrations in cats. The experiment was designed to titrate equivalent levels of DHA in the feline foods to establish DHA bioequivalency from each source.

## Materials and Methods

All experimental procedures were reviewed and approved by the Hill’s Pet Nutrition Institutional Animal Care and Use Committee (protocol CP997) and were in accordance with Hill’s Global Animal Welfare Policy. At no time were animals subjected to any procedures expected to cause pain or distress.

### Animals and experimental design

Healthy adult cats under the age of 13 yr that would be suitable to consume dry food for the duration of the study were included. Cats were excluded from the study if they had been diagnosed with a systemic illness, had known gastrointestinal or skin sensitivities, were pregnant, had a planned surgery, were taking n-3 or vitamin supplements, or were fractious. Cats could subsequently be removed from the study if they experienced weight loss or gain above 15%, stopped eating for 3 d, or were diagnosed with any secondary systemic disease as described in the exclusion criteria above.

All cats were group housed and maintained at the Hill’s Pet Nutrition Center and treated in accordance with Hill’s Global Animal Welfare Policy. Once enrolled, cats had access to electronic feeders and were fed fresh food once daily to maintain ideal body weight. Food intake was recorded daily for each cat. Feeders allowed access to food until individuals had consumed the amount calculated for body weight maintenance. Water was offered ad libitum. Cats were housed in a temperature-controlled facility with access to natural light that varied with seasonal changes. Cats had access to toys and experienced daily enrichment that provided opportunities for them to engage in activities to satisfy their natural behaviors and instincts, as well as socialization to ensure they were comfortable and happy. The study design did not interfere with their normal daily routine.

**Figure 1.**
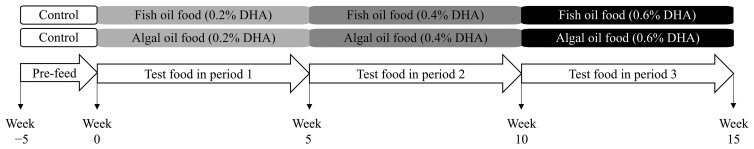
Study design and timeline. Cats underwent a 5-wk period of being fed a control food prior to being randomized to the fish oil food or algal oil food groups. Groups were sequentially fed foods that contained either fish oil or algal oil at concentrations formulated with 0.2, 0.4, or 0.6% dietary DHA for 5 wk per food with no washout between periods. Body weight measurements were taken and blood was collected on day 1 and at the end of each 5-wk period.

Twenty-six healthy adult male and female cats were split into two groups in a two-arm, completely randomized design: group 1 (fish oil foods) and group 2 (algal oil foods). Both groups were fed a control food during the pre-feed period of 5 wk. The control food was formulated to avoid ingredients that contain EPA or DHA in order to help establish a baseline serum concentration of these fatty acids. For the remainder of the study, the groups were fed foods that contained either fish oil or algal oil at concentrations designed to deliver 0.2, 0.4, and 0.6% dietary DHA for 5 wk per food in a sequential design with no washout between periods (Figure 1).

Body weight was measured on day 1 and at the end of each 5-wk period. Food intake was recorded daily. Blood samples were collected via jugular venipuncture, and collections for serum fatty acid analysis were obtained on day 1 and at the end of each 5-wk period.

### Study foods

All study foods were formulated using Concept5 (CFC Tech Services, Inc., Pierz, MN, USA). All foods were designed to deliver similar macronutrient and metabolizable energy levels. To ensure that study foods were isocaloric, other fat sources in the foods were adjusted to account for the addition of fish or algal oil. The nutrient analyses of the finished study foods were performed by Eurofins (Des Moines, IA, USA) using official methods published by AOAC International (AOAC International, 2019). The nutrient compositions and ingredients of the study foods are summarized in Tables 1 and 2. The fish oil was from fish species containing high amounts of n-3 fatty acids, such as anchovy, mackerel, and sardine (Lysi, Reykjavik, Iceland). Algal oil is a nutritional oil from the marine algae *Schizochytrium* sp., a rich source of EPA and DHA (Veramaris, Delft, Netherlands). Compositions of the oils and their peroxide values are listed in Table 3.

**Table 1.** Nutrient composition on an as-fed basis of test foods containing 0, 0.2, 0.4, and 0.6% docosahexaenoic acid from either fish oil or algal oil

	Treatment						
Nutrient	Control	Fish oil food			Algal oil food		
DHA	0%	0.2%	0.4%	0.6%	0.2%	0.4%	0.6%
Metabolizable energy, kcal/kg^1^	4171	4246	4208	4168	4208	4175	4167
Moisture	5.71	5.59	5.17	5.08	5.86	5.60	5.65
Ash	4.34	4.25	4.30	4.18	4.09	4.24	4.33
Crude protein	27.00	27.94	27.81	27.69	27.00	27.63	27.38
Crude fat	21.09	22.30	21.35	20.41	21.69	20.88	20.95
Crude fiber	0.90	0.70	0.80	0.80	0.80	0.70	0.90
Total n-3 fatty acids^2^	0.18	0.91	1.43	2.08	0.49	0.78	1.15
Total n-6 fatty acids^3^	3.86	3.95	3.61	3.34	3.86	3.73	3.82
n-6:n-3 ratio	21.44:1	4.34:1	2.52:1	1.61:1	7.88:1	4.78:1	3.32:1
C18:2 LA	3.67	3.70	3.35	3.05	3.64	3.49	3.55
C18:3 ALA	0.17	0.19	0.20	0.19	0.17	0.16	0.17
C20:4 ARA	0.08	0.12	0.14	0.18	0.10	0.12	0.14
C20:5 EPA	<0.02	0.35	0.60	0.92	0.10	0.19	0.30
C22:6 DHA	<0.02	0.23	0.39	0.60	0.19	0.38	0.60

Cats were sequentially fed the fish oil or algal oil foods for 5-wk periods. Values are g/100 g unless otherwise indicated.

ALA, alpha-linolenic acid; ARA, arachidonic acid; DHA, docosahexaenoic acid; EPA, eicosapentaenoic acid; LA, linoleic acid.

^1^Calculated using modified Atwater coefficients (Jewell and Jackson, 2023).

^2^Sum of C18:3, C18:4, C20:3, C20:4, C20:5, C21:5, C22:3, C22:5, C22:6.

^3^Sum of C18:2, C18:3, C20:2, C20:3, C20:4, C22:2, C22:4, C22:5.

**Table 2.** Ingredients of the study foods containing 0, 0.2, 0.4, and 0.6% docosahexaenoic acid from either fish oil or algal oil

	Treatment						
	Control	Fish oil			Algal oil		
DHA	0%	0.2%	0.4%	0.6%	0.2%	0.4%	0.6%
**Ingredient, %**							
Brewer’s rice	39.90	39.90	39.90	39.90	39.90	39.90	39.90
Corn protein meal	17.97	17.97	17.97	17.97	17.97	17.97	17.97
Dried chicken	7.00	7.00	7.00	7.00	7.00	7.00	7.00
Beet pulp	3.50	3.50	3.50	3.50	3.50	3.50	3.50
Dried egg	3.50	3.50	3.50	3.50	3.50	3.50	3.50
Soybean protein isolate	3.50	3.50	3.50	3.50	3.50	3.50	3.50
Mineral premix	2.64	2.64	2.64	2.64	2.64	2.64	2.64
Carnitine	0.55	0.55	0.55	0.55	0.55	0.55	0.55
l-arginine monohydrochloride	0.50	0.50	0.50	0.50	0.50	0.50	0.50
d,l-methionine	0.40	0.40	0.40	0.40	0.40	0.40	0.40
Taurine	0.26	0.26	0.26	0.26	0.26	0.26	0.26
Vitamin premix	0.25	0.25	0.25	0.25	0.25	0.25	0.25
l-lysine hydrochloride	0.33	0.33	0.33	0.33	0.33	0.33	0.33
l-threonine	0.23	0.23	0.23	0.23	0.23	0.23	0.23
l-tryptophan	0.06	0.06	0.06	0.06	0.06	0.06	0.06
Lactic acid blend	1.20	1.20	1.20	1.20	1.20	1.20	1.20
Choline chloride	0.44	0.44	0.44	0.44	0.44	0.44	0.44
Refined chicken fat	15.65	13.65	11.65	9.65	15.12	14.58	14.04
Fish oil	0	2.00	4.00	6.00	0	0	0
Algal oil	0	0	0	0	0.54	1.07	1.61
Palatant	2.50	2.50	2.50	2.50	2.50	2.50	2.50

Cats were sequentially fed the fish oil or algal oil foods for 5-wk periods. Values shown are percent of total food.

**Table 3.** Composition of the fish and algal oils used in the study foods

	Fish oil	Algal oil
Moisture	0	0
Total n-3 fatty acids^1^	31.3	58.1
Total n-6 fatty acids^2^	3.0	5.8
C18:2 LA	1.2	0
C18:3 ALA	0.5	0.1
C20:4 ARA	1.6	3.7
C20:5 EPA	15.3	17.7
C22:6 DHA	9.8	36.1
Peroxide value, meg/kg	4.5	0.2

Values are % unless otherwise indicated.

ALA, alpha-linolenic acid; ARA, arachidonic acid; DHA, docosahexaenoic acid; EPA, eicosapentaenoic acid; LA, linoleic acid.

^1^Sum of C18:3, C18:4, C20:3, C20:4, C20:5, C21:5, C22:3, C22:5, C22:6.

^2^Sum of C18:2, C18:3, C20:2, C20:3, C20:4, C22:2, C22:4, C22:5.

### Analyses of blood samples

Serum EPA, DHA, linoleic acid, alpha-linolenic acid, and arachidonic acid were measured via liquid chromatography/quadrupole time-of-flight (LC/Q-TOF) at Hill’s Pet Nutrition using a modification of a previously published method (Serafim et al., 2019). Serum samples were incubated under basic conditions at 80 °C for 30 min to convert chemically and physically bonded fatty acids to free fatty acids. After neutralizing with dilute formic acid solution, samples were centrifuged at 4,450 × *g* for 10 min to precipitate any particulate material. The concentrations of fatty acids in serum samples were then quantified with LC/Q-TOF.

### Statistical analysis

Body weight data were analyzed using a linear mixed model with oil type, oil concentration, and the interaction as fixed effects and animal as a random effect. The two oil types were compared to each other at each oil concentration using single degree-of-freedom (df) estimate statements. The Kenward-Roger adjustment (DDFM = KR) was used to adjust the denominator df in the *F*-test and the standard errors of the means for the presence of multiple random effects in the model. The analysis was performed using PROC MIXED in SAS, version 9.4 (Cary, NC, USA). Intake (both grams and calories) was analyzed using the same model described above for body weight, using the mean intake for each animal on each food.

Serum fatty acid data were analyzed using a random coefficient model with oil concentration in the food as a continuous covariate (Brown and Prescott, 2006). Random intercepts and slopes were estimated using the RANDOM option. The NOBOUND option was used to allow for negative covariance estimates between the intercept and slope estimates. Both linear and quadratic models were evaluated for each fatty acid. However, if the quadratic term was statistically not significant, it was dropped, and linear trends over oil concentration were chosen as the final model. DDFM = KR was used to estimate the denominator degrees of freedom in the *F*-tests. Separate models were fit for each oil type. The analysis was performed using PROC GLIMMIX in SAS, version 9.4. Graphs showing trends over each oil concentration were generated for each serum fatty acid.

## Results

### Population

Twenty-six cats were enrolled, and 24 completed the study. The distribution of sex, as well as initial body weight and age, was similar between cats who consumed the fish oil or algal oil test foods (Table 4). Both foods were similarly well tolerated. Two cats were removed from the study: one due to a diagnosis of early onset renal failure (algal oil group) and the other due to an injury that required treatment (fish oil group). These events were assessed to be unrelated to the study foods.

**Table 4.** Cat characteristics at study baseline prior to being sequentially fed foods containing 0.2, 0.4, and 0.6% docosahexaenoic acid from either fish oil or algal oil for 5-wk periods

	Test food		
Characteristic	Fish oil food	Algal oil food	*P* value
Animals, *n*	13	13	-
Neutered male	7 (47)	8 (55)	-
Spayed female	6 (53)	5 (45)	-
Age, y	7.53 ± 2.91	6.94 ± 2.26	0.5662
Body weight, kg	5.38 ± 0.94	5.45 ± 0.76	0.8269
Serum DHA, mg/dL	2.72 ± 0.91	3.09 ± 1.35	0.4160

Data are *n* (%) or means ± standard error.

### Food intake

**Figure 2.**
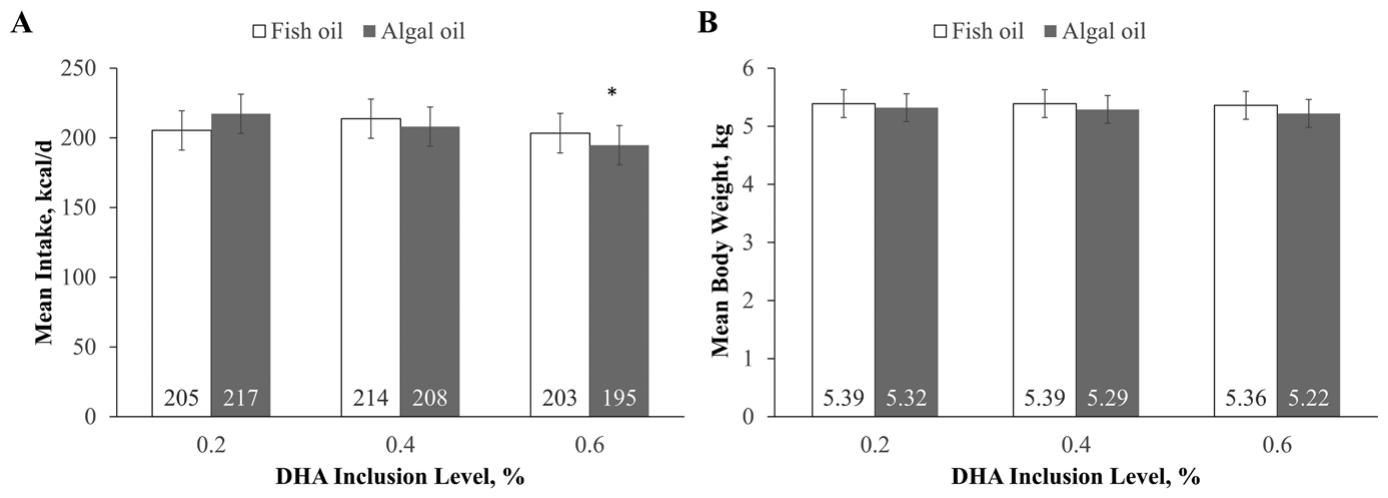
(A) Mean intakes and (B) body weights in cats sequentially fed the fish oil or algal oil foods with DHA at 0.2, 0.4, or 0.6% for 5 wk. Data are means ± standard error. Mean values are indicated within the bars. *P = 0.0011 from comparison of intake (kcal) during baseline vs. during provision of algal oil food with DHA at 0.6%.

There was no significant difference in kilocalories (Figure 2) or grams (data not shown) consumed on a dry matter or as-fed basis per day among cats fed foods containing algal oil or fish oil except that intake was significantly lower in cats fed the algal oil food with 0.6% DHA compared with the baseline (control) value (*P* = 0.0011).

### Body weight

Oil type had no effect on body weight at the end of each 5-wk period between cats consuming either algal or fish oil (Figure 2B). Although oil level had a significant effect (*P* = 0.019) on cats’ body weights, the effect was small (a decrease of 0.07 kg at the highest oil level) and was not considered to be clinically significant. Similarly, body weights varied significantly (*P *= 0.0325) among weeks, but there was no evidence of a clinically meaningful pattern.

### Serum fatty acid concentrations

**Figure 3.**
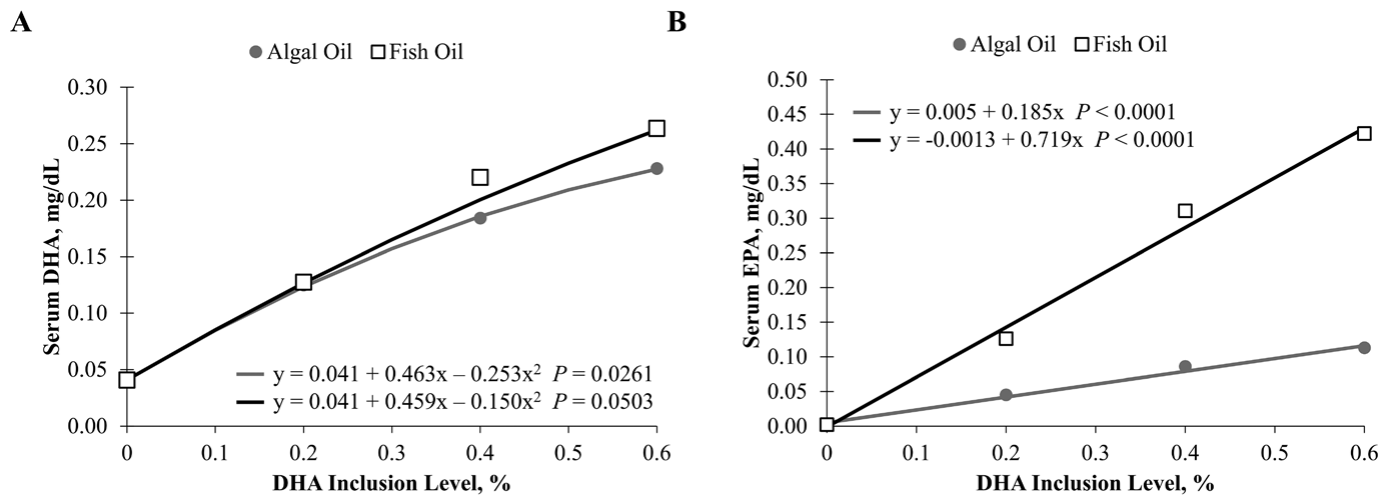
Serum fatty acid levels for (A) DHA and (B) EPA in cats sequentially fed the fish oil or algal oil foods with DHA at 0.2, 0.4, or 0.6% for 5 wk. Serum DHA was 0.04, 0.12, 0.18, and 0.23 mg/dL for algal oil at DHA inclusion levels of 0, 0.2, 0.4, and 0.6%, respectively, and was 0.04, 0.13, 0.22, and 0.26 mg/dL for fish oil at DHA inclusion levels of 0, 0.2, 0.4, and 0.6%, respectively. Serum EPA was 0, 0.05, 0.09, and 0.11 mg/dL for algal oil at DHA inclusion levels of 0, 0.2, 0.4, and 0.6%, respectively, and was 0, 0.13, 0.31, and 0.42 mg/dL for fish oil at DHA inclusion levels of 0, 0.2, 0.4, and 0.6%, respectively. Fit equations and *P* values are shown in their respective graphs.

**Figure 4.**
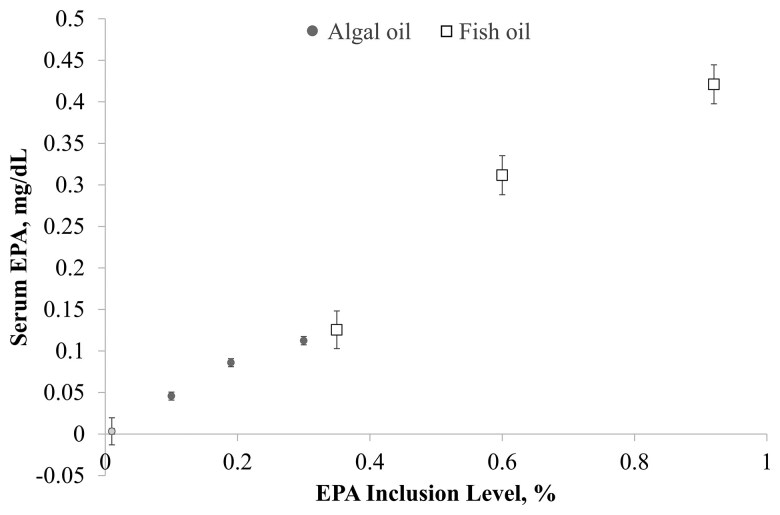
Serum EPA levels by the EPA percentage in the food in cats sequentially fed the fish oil or algal oil foods with DHA at 0.2, 0.4, or 0.6% for 5 wk.

**Figure 5.**
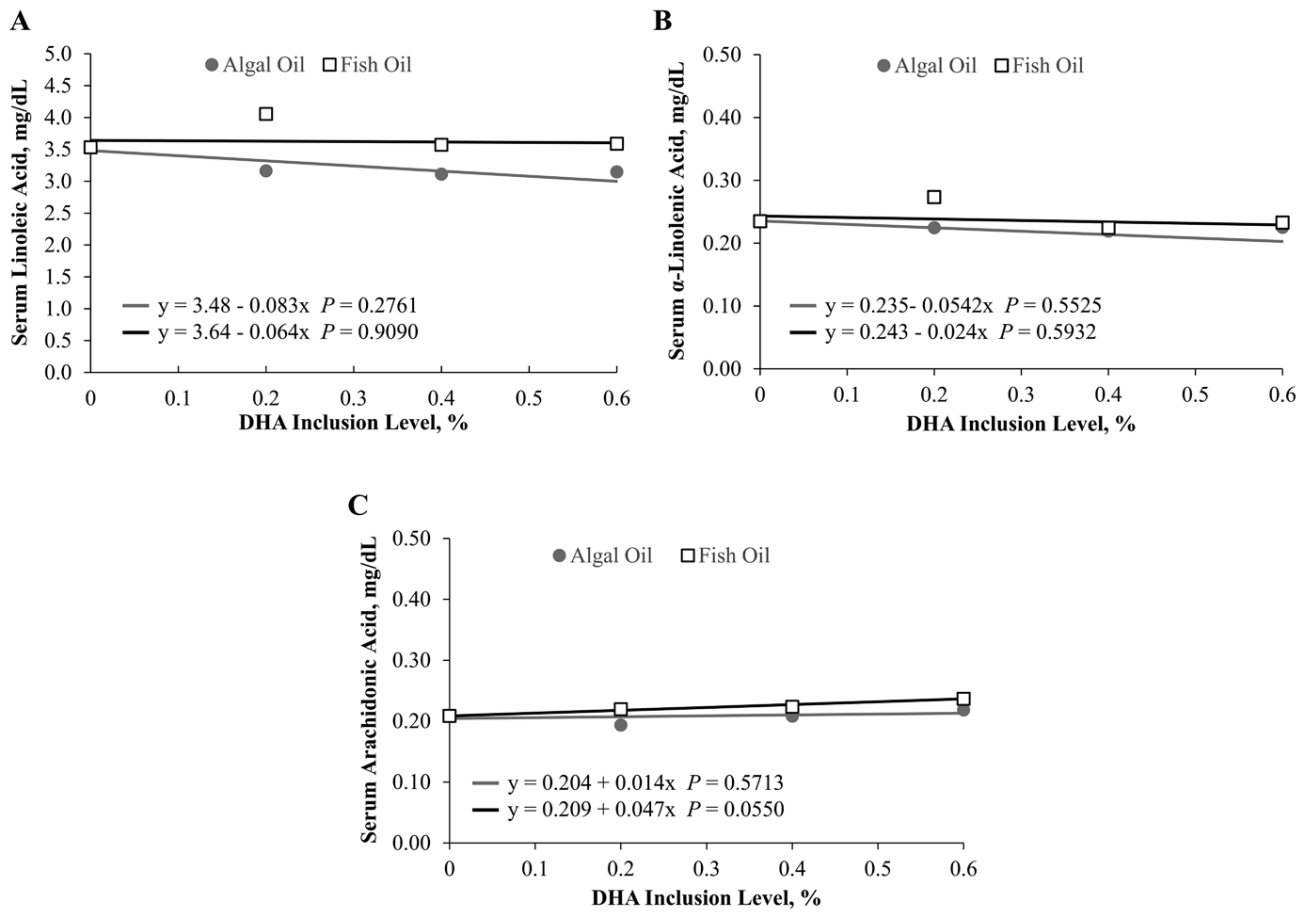
Serum fatty acid levels for (A) linoleic acid, (B) alpha-linolenic acid, and (C) arachidonic acid in cats sequentially fed the fish oil or algal oil foods with DHA at 0.2, 0.4, or 0.6% for 5 wk.

Increasing concentrations of fish and algal oils in the foods led to increases in serum DHA at similar rates. That is, the slopes of the lines are similar, and the significant fit (*P* ≤ 0.05) indicates that as DHA increased in food, serum DHA levels significantly changed as well (Figure 3A). Serum EPA concentrations also increased as fish oil and algal oil levels increased in the food (Figure 3B; Figure 4). Increasing fish and algal oil levels had no significant effect on serum linoleic acid, alpha-linolenic acid, or arachidonic acid concentrations (Figure 5) despite the increase in dietary arachidonic acid concentrations with increasing fish oil or algal oil in the foods.

## Discussion

This study evaluated the effects of replacing fish oil with algal oil in feline foods. Alternatives to fish oil are being sought since worldwide demands for fish oil are outpacing the production of fisheries (Jenkins et al., 2009; Oils and Fats International, 2024). Inclusion of fish oil and algal oil in the study foods had no effect on body weight. An effect on intake was only observed in cats fed the algal oil food with 0.6% DHA compared with baseline, which may indicate an effect on palatability at this higher level, though additional study would be needed. Otherwise, both oils appeared to be well tolerated and palatable.

Current data are insufficient for establishing an adult maintenance minimum or maximum levels for EPA and DHA, but AAFCO noted that an argument could be made to recommend 0.02% EPA + DHA on a dry matter basis as a minimum in adult cats based on their low delta-6 desaturase activity (AAFCO, 2024). AAFCO recommends 0.012% EPA + DHA on a dry matter basis as a minimum for cats for growth and reproduction based on the requirement of these n-3 PUFAs during fetal and neonatal development of vision and the nervous system (AAFCO, 2024). Similarly, FEDIAF recommends 0.01% EPA + DHA on a dry matter basis for growth and reproduction in cats (European Pet Food Industry Federation (FEDIAF), 2024). Of note, the lowest level of EPA + DHA used in this study was 0.31% on a dry matter basis (present in the food with algal oil with 0.2% DHA [0.29% on an as-fed basis]). The highest level of EPA + DHA used in this study was 1.60% on a dry matter basis (present in the food with fish oil with 0.6% DHA [1.52% on an as-fed basis]), which is similar to the 1.62% that was also found to be well tolerated in cats during mating, gestation, lactation, and growth (Vuorinen et al., 2020).

Notably, the concentrations of EPA and DHA used here are similar to some of those used in prior studies that found positive effects on health in cats with fish oil supplementation. The 1.88 g/1,000 kcal EPA + DHA food, fed for 9 wk, that improved mobility in cats with degenerative joint disease (Lascelles et al., 2010) is similar to the amount of EPA + DHA in the algal oil food with 0.6% DHA used in the present study (2.1 g/1,000 kcal). In addition, the food with 0.09% EPA and 0.18% DHA (both on an as-fed basis) fed to cats for 8 wk in a study that found a reduced risk of urinary stone formation (Hall et al., 2017) had similar amounts of these PUFAs as in the algal oil food with 0.2% DHA used in the present study (0.10% EPA, 0.19% DHA). It will be interesting to test these foods for beneficial health effects in future studies.

As in the studies that tested dietary supplementation with several levels of algal oil in cats and dogs during gestation, lactation, and growth (Dahms et al., 2019; Vuorinen et al., 2020), levels of serum EPA and DHA increased in the present study in a dose-dependent manner. These results are also consistent with those of a human study in which DHA levels in plasma phospholipids and erythrocytes increased in a dose-dependent and linear manner with dietary algal oil supplementation (Arterburn et al., 2007). However, in the present study, serum EPA increased to a greater extent with higher DHA inclusion in the fish oil supplementation than with algal oil supplementation. This result was not unexpected, given that the ratio of EPA:DHA in the oils used in this study is approximately 1.5:1 in the fish oil but is 0.38:1 in the algal oil, and because of this, fish oil inclusion was 3.7-fold higher than algal oil inclusion in the foods. Thus, because the foods were formulated to contain equivalent concentrations of DHA, EPA concentrations were 3.1- to 3.5-fold higher (depending on the food) in the fish oil foods than in the algal oil foods. Plotting serum EPA by dietary EPA shows that both oils led to increases in serum EPA at similar rates (see Figure 4). Further, a 12-wk study in humans found that there is no retroconversion of DHA to EPA but that EPA substantially converts to DHA upon supplementation with EPA (Metherel et al., 2019), so assuming that this is also true in cats, the serum EPA concentration is a reflection of the dietary EPA and not dietary DHA. Additional EPA could serve as a precursor to eicosanoids or resolvins (Calder, 2013). The difference in the fatty acid profiles of the fish and algal oils used in the present study warrants further research to fully understand the impact on clinical health when different EPA and DHA concentrations are fed.

In this study, no significant effect was observed on serum linoleic acid, alpha-linolenic acid, or arachidonic acid concentrations with increasing fish and algal oil levels. However, there were small increases in dietary arachidonic acid concentrations with increasing fish oil or algal oil in the foods. Perhaps these increases in dietary arachidonic acid were not great enough to confer an observable change in serum concentrations of arachidonic acid. Alternatively, the arachidonic acid could have been used in eicosanoid production (Whelan et al., 1997).

## Conclusions

Overall, these data indicate that algal oil may serve as a good dietary source of DHA and as an alternative to fish oil in feline foods. The algal oil utilized in the present study contains approximately 3 to 3.5-fold more DHA than fish oil. However, further research is necessary to understand the clinical application of a high-DHA, lower-EPA algal oil.

## Abbreviations

AAFCO: American Association of Feed Control Officials
DHA: docosahexaenoic acid
EPA: eicosapentaenoic acid
PUFAs: polyunsaturated fatty acids
